# Adrenaline autoinjector is under-prescribed in typical cold urticaria patients living in tropical climate countries

**DOI:** 10.5339/qmj.2022.fqac.19

**Published:** 2022-03-24

**Authors:** Mojca Bizjak, Mitja Košnik, Dejan Dinevski, Simon Francis Thomsen, Daria Fomina, Elena Borzova, Kanokvalai Kulthanan, Raisa Meshkova, FernandoM Aarestrup, Dalia Melina Ahsan, Mona Al-Ahmad, Sabine Altrichter, Andrea Bauer, Maxi Brockstädt, Célia Costa, Semra Demir, Roberta Fachini Criado, Luis Felipe Ensina, Asli Gelincik, Ana Maria Giménez-Arnau, Margarida Gonçalo, Maia Gotua, Jesper Grønlund Holm, Naoko Inomata, Alicja Kasperska-Zajac, Maryam Khoshkhui, Aliya Klyucharova, Emek Kocatürk, Rongbiao Lu, Michael Makris, Natalya Maltseva, Maria Pasali, Marisa Paulino, David Pesqué, Jonny Peter, German Dario Ramón, Carla Ritchie, Solange Oliveira Rodrigues Valle, Michael Rudenko, Agnieszka Sikora, Nicola Wagner, Paraskevi Xepapadaki, Xiaoyang Xue, Zuotao Zhao, Dorothea Terhorst-Molawi, Marcus Maurer

**Affiliations:** ^1†*^COLD-CE steering committee member, ^*^both authors have contributed equally; ^1^Division of Allergy, Urticaria Center of Reference and Excellence (UCARE), University Clinic of Respiratory and Allergic Diseases Golnik, Golnik, Slovenia E-mail: mojca.bizjak@klinika-golnik.si; ^2^Faculty of Medicine, University of Maribor, Maribor, Slovenia; ^3^Faculty of Medicine, University of Ljubljana, Ljubljana, Slovenia; ^4^Department of Dermatology, Urticaria Center of Reference and Excellence (UCARE), Bispebjerg Hospital, University of Copenhagen, Copenhagen, Denmark; ^5^Center of Allergy and Immunology, Urticaria Center of Reference and Excellence (UCARE), Clinical State Hospital 52, Moscow Ministry of Healthcare, Moscow, Russian Federation; ^6^Department of Clinical Immunology and Allergology, I.M. Sechenov First Moscow State Medical University, Moscow, Russian Federation; ^7^Department of Dermatology and Venereology, I.M. Sechenov First Moscow State Medical University, Moscow, Russian Federation; ^8^Department of Clinical Genetics, Veltischev Research and Clinical Institute for Pediatrics of the Pirogov Russian National Research Medical University, Moscow, Russian Federation; ^9^Department of Dermatology, Urticaria Center of Reference and Excellence (UCARE), Faculty of Medicine Siriraj Hospital, Mahidol University, Bangkok, Thailand; ^10^Department of Clinical Immunology and Allergology, Urticaria Center of Reference and Excellence (UCARE), Smolensk State Medical University, Smolensk, Russian Federation; ^11^Faculdade de Ciências, Médicas e da Saúde de Juiz de Fora (SUPREMA), Urticaria Center of Reference and Excellence (UCARE), Hospital Maternidade Therezinha de Jesus, Minas Gerais, Brazil; ^12^Urticaria Center of Reference and Excellence (UCARE), Institute of Allergology, Charité –Universitätsmedizin Berlin, corporate member of Freie Universität Berlin and Humboldt-Universität zu Berlin, Berlin, Germany; ^13^Fraunhofer Institute for Translational Medicine and Pharmacology ITMP, Allergology and Immunology, Berlin, Germany; ^14^Microbiology Department, Faculty of Medicine, Urticaria Center of Reference and Excellence (UCARE), Kuwait University, Safat, Kuwait; ^15^Department of Dermatology and Venerology, Urticaria Center of Reference and Excellence (UCARE), Comprehensive Allergy Center, Kepler University Hospital, Linz, Austria; ^16^Department of Dermatology, Urticaria Center of Reference and Excellence (UCARE), University Allergy Center, University Hospital Carl Gustav Carus, Technical University, Dresden, Germany; ^17^Immunoallergology Department, Urticaria Center of Reference and Excellence (UCARE), Hospital de Santa Maria, CHULN, Lisbon, Portugal; ^18^Division of Allergy, Department of Internal Medicine, Istanbul Faculty of Medicine, Urticaria Center of Reference and Excellence (UCARE), Istanbul University, Istanbul, Turkey; ^19^Faculdade de Medicina do ABC (FMABC), Urticaria Center of Reference and Excellence (UCARE), Santo André, Brazil; ^20^Division of Allergy, Clinical Immunology and Rheumatology, Department of Pediatrics, Urticaria Center of Reference and Excellence (UCARE), Federal University of São Paulo, São Paulo, Brazil; ^21^Department of Dermatology, Urticaria Center of Reference and Excellence (UCARE), Hospital del Mar, IMIM, Universitat Autònoma, Barcelona, Spain; ^22^Clinic of Dermatology, Urticaria Center of Reference and Excellence (UCARE), University Hospital and Faculty of Medicine, University of Coimbra, Coimbra, Portugal; ^23^Center of Allergy and Immunology, Urticaria Center of Reference and Excellence (UCARE), Tbilsi, Georgia; ^24^Department of Environmental Immuno-Dermatology, Urticaria Center of Reference and Excellence (UCARE), Yokohama City University Graduate School of Medicine, Yokohama, Japan; ^25^European Center for Diagnosis and Treatment of Urticaria/Angioedema (GA2LEN UCARE /ACARE Network) & Department of Clinical Allergology and Urticaria of Medical University of Silesia, Poland; ^26^Allergy Research Center, Mashhad University of Medical Sciences, Mashhad, Iran; ^27^Department of Clinical Immunology and Allergology, Republican Center of Clinical Immunology and Allergology, Urticaria Center of Reference and Excellence (UCARE), Republican Clinical Hospital, Kazan State Medical University, Kazan, Russian Federation; ^28^Department of Internal Diseases, Institute of Fundamental Medicine and Biology (IFMB) of Kazan Federal University, Kazan, Russian Federation; ^29^Department of Dermatology, Urticaria Center of Reference and Excellence (UCARE), Koç University School of Medicine, Istanbul, Turkey; ^30^Department of Dermatology, Urticaria Center of Reference and Excellence (UCARE), The Third Affiliated Hospital of Sun Yat-sen University, Guangzhou, China; ^31^Allergy Unit, Second Department of Dermatology and Venereology, Urticaria Center of Reference and Excellence (UCARE), National and Kapodistrian University of Athens, University General Hospital “Attikon”, Athens, Greece; ^32^Division of Allergy and Clinical Immunology, Department of Medicine, Urticaria Center of Reference and Excellence (UCARE), University of Cape Town, Cape Town, South Africa; ^33^Allergy and Immunology Unit, University of Cape Town Lung Institute, Cape Town, South Africa; ^34^Instituto de Alergia e Inmunologia del Sur, Urticaria Center of Reference and Excellence (UCARE), Buenos Aires, Argentina; ^35^Adults and Pediatrics Allergy Unit, Urticaria Center of Reference and Excellence (UCARE), Hospital Italiano de Buenos Aires, Buenos Aires, Argentina; ^36^Department of Internal Medicine, Immunology Service, Urticaria Center of Reference and Excellence (UCARE), Federal University of Rio de Janeiro, Rio de Janeiro, Brazil; ^37^London Allergy and Immunology Centre, Urticaria Center of Reference and Excellence (UCARE), London, UK; ^38^Department of Dermatology, Urticaria Center of Reference and Excellence (UCARE), University Hospital of Erlangen, University of Erlangen-Nuremberg (FAU), Germany; ^39^Allergy Unit, 2nd Pediatric Clinic, Urticaria Center of Reference and Excellence (UCARE), National and Kapodistrian University of Athens, Greece; ^40^Department of General Practice, Urticaria Center of Reference and Excellence (UCARE), Community Health Service Center, Guangzhou City, China; ^41^Department of Dermatology and Venerology, Urticaria Center of Reference and Excellence (UCARE), Beijing Key Laboratory of Molecular Diagnosis on Dermatoses and National Clinical Research Center for Skin and Immune Diseases, Peking University First Hospital, Beijing, China

**Keywords:** **Keyword:** AAI, ColdU, ColdA

## Abstract

**Background:** The diagnosis of typical cold urticaria (ColdU) relies on whealing in response to local cold stimulation testing (CST). It can also manifest with cold-induced anaphylaxis (ColdA). Till date, it is largely unclear how often patients with ColdU receive adrenaline treatment and are provided with an adrenaline autoinjector (AAI). **Methods:** An international, cross-sectional study, COLD-CE (i.e., comprehensive evaluation of ColdU and other cold-induced reactions), was carried out at 32 UCAREs. Detailed histories were taken and CST with an ice cube and/or TempTest^®^ performed. ColdA was defined as an acute cold-induced (i.e., by cold water, air, or surfaces) involvement of the skin and/or visible mucosal tissue and at least one of the symptoms (cardiovascular manifestations, difficulty breathing, or gastrointestinal symptoms). **Results:** Of the 551 ColdU patients, 75% (*n* = 412) had a positive CST. Of them, concomitant chronic spontaneous urticaria was diagnosed in 10%. Of 372 patients with stand-alone ColdU, 69% were women and 91% adults. Their median age was 36 (IQR 26 − 48) years. Patients were also categorized into residents of countries with a tropical (*n* = 33), temperate (*n* = 264), or cold (*n* = 75) climate (Table 1: R13C1, R17C1, R21C1). AAI was more often prescribed to residents of temperate than tropical countries (30% vs. 12%, *p* = .038; Table 1: R31C1), although the frequency of ColdA did not significantly differ between these countries (44% vs. 42%, *p* = 1.000; R29C2). Residents of tropical countries had a higher frequency of ColdA induced by cold air than residents of temperate (36% vs. 12%, *p* = .001; R29C4) or cold (36% vs. 12%, *p* = .007; R25C4) countries. Cardiovascular manifestations induced by cold air were diagnosed in 33% (*n* = 11) of residents of tropical countries, but only 18% (*n* = 2) and 36% (*n* = 4) of them had received adrenaline and AAI, respectively (R13 − 15C7). Furthermore, hypotension and/or loss of consciousness induced by cold air occurred in 18% (*n* = 6) of patients, but only 17% (*n* = 1) received adrenaline (R13 − 14C10). ColdA was induced by complete cold water immersion in 9% (*n* = 3) of patients, and none of them received adrenaline treatment nor AAI (R13 − 15C3). **Conclusion:** Our findings suggest that ColdA is undertreated and call for changes in ColdU management.

## Figures and Tables

**Table 1:** Previous adrenaline treatment, AAI prescription or both stratified by systemic reactions in typical ColdU patients.

C1	C2	C3	C4	C5	C6	C7	C8	C9	C10
		ColdU	ColdA – broad definition			Cardiovascular manifestations			Hypotension and/or loss of consciousness		
			Any^a^	CWI	Air	Any^a^	CWI	Air	Any^a^	CWI	Air
R1	Mixed adult/pediatric	N=372	145 (39%)	107 (29%)	53 (14%)	116 (31%)	88 (24%)	31 (8%)	48 (13%)	41 (11%)	10 (3%)
R2	Adrenaline received^b^	12 (3%)	12 (8%)	8 (8%)	4 (8%)	11 (10%)	7 (8%)	4 (13%)	8 (17%)	5 (12%)	3 (30%)
R3	AAI prescribed^b^	93 (25%)	54 (37%)	44 (41%)	15 (28%)	49 (42%)	41 (47%)	9 (29%)	24 (50%)	21 (51%)	4 (40%)
R4	Both^c^	8 (2%)	8 (6%)	5 (5%)	3 (6%)	7 (6%)	4 (5%)	3 (10%)	5 (10%)	3 (7%)	2 (20%)
R5	Adult (≥18 years)	N=338	135 (40%)	98 (29%)	51 (15%)	106 (31%)	79 (23%)	29 (9%)	41 (12%)	35 (10%)	9 (3%)
R6	Adrenaline received^b^	10 (3%)	10 (7%)	7 (7%)	3 (6%)	9 (9%)	6 (8%)	3 (10%)	6 (15%)	4 (11%)	2 (22%)
R7	AAI prescribed^b^	85 (25%)	51 (38%)	42 (43%)	14 (28%)	46 (43%)	39 (49%)	8 (28%)	22 (54%)	20 (57%)	3 (33%)
R8	Both^c^	7 (2%)	7 (5%)	5 (5%)	2 (4%)	6 (6%)	4 (5%)	2 (7%)	4 (10%)	3 (9%)	1 (11%)
R9	Pediatric	N=34	10 (29%)	9 (27%)	2 (6%)	10 (29%)	9 (27%)	2 (6%)	7 (21%)	6 (18%)	1 (3%)
R10	Adrenaline received^b^	2 (6%)	2 (20%)	1 (11%)	1 (50%)	2 (20%)	1 (11%)	1 (50%)	2 (29%)	1 (17%)	1 (100%)
R11	AAI prescribed^b^	8 (24%)	3 (30%)	2 (22%)	1 (50%)	3 (30%)	2 (22%)	1 (50%)	2 (29%)	1 (17%)	1 (100%)
R12	Both^c^	1 (3%)	1 (10%)	0	1 (50%)	1 (10%)	0	1 (50%)	1 (14%)	0	1 (100%)
R13	Tropical climate	N=33	14 (42%)	3 (9%)	12 (36%)	12 (36%)	1 (3%)	11 (33%)	7 (21%)	3 (9%)	6 (18%)
R14	Adrenaline received^b^	2 (6%)	2 (14%)	0	2 (17%)	2 (17%)	0	2 (18%)	1 (14%)	0	1 (17%)
R15	AAI prescribed^b^	4 (12%)	4 (29%)	0	4 (33%)	4 (33%)	0	4 (36%)	3 (43%)	1 (33%)	3 (50%)
R16	Both^c^	2 (6%)	2 (14%)	0	2 (17%)	2 (17%)	0	2 (18%)	1 (14%)	0	1 (17%)
R17	Temperate climate	N=264	115 (44%)	94 (36%)	32 (12%)	95 (36%)	80 (30%)	18 (7%)	38 (14%)	35 (13%)	3 (1%)
R18	Adrenaline received^b^	10 (4%)	10 (9%)	8 (9%)	2 (6%)	9 (10%)	7 (9%)	2 (11%)	7 (18%)	5 (14%)	2 (67%)
R19	AAI prescribed^b^	78 (30%)	46 (40%)	41 (44%)	10 (31%)	42 (44%)	38 (48%)	5 (28%)	20 (53%)	19 (54%)	1 (33%)
R20	Both^c^	6 (2%)	6 (5%)	5 (5%)	1 (3%)	5 (5%)	4 (5%)	1 (6%)	4 (11%)	3 (9%)	1 (33%)
R21	Cold climate	N=75	16 (21%)	10 (13%)	9 (12%)	9 (12%)	7 (9%)	2 (3%)	3 (4%)	3 (4%)	1 (1%)
R22	Adrenaline received^b^	0	0	0	0	0	0	0	0	0	0
R23	AAI prescribed^b^	11 (15%)	4 (25%)	3 (30%)	1 (11%)	3 (33%)	3 (43%)	0	1 (33%)	1 (33%)	0
R24	Both^c^	0	0	0	0	0	0	0	0	0	0
R25	Tropical vs. cold (p-value)		.035	.750	.007	.007	.430	**<**.001	.009	.367	.003
R26	Adrenaline received^b^	.091	.209	NA	.486	.486	NA	1.000	1.000	NA	1.000
R27	AAI prescribed^b^	1.000	1.000	.528	.338	1.000	1.000	1.000	1.000	1.000	1.000
R28	Both^c^	.091	.209	NA	.486	.486	NA	1.000	1.000	NA	1.000
R29	Tropical vs. temperate (*p*-value)		1.000	.001	.001	1.000	**<**.001	**<**.001	.306	.781	**<**.001
R30	Adrenaline received^b^	.630	.619	1.000	.297	.357	1.000	.622	1.000	1.000	.226
R31	AAI prescribed^b^	.038	.564	.260	1.000	.549	1.000	.694	.699	.595	1.000
R32	Both^c^	.219	.210	1.000	.176	.176	1.000	.539	1.000	1.000	1.000
R33	Temperate vs. cold (p-value)		**<**.001	**<**.001	1.000	**<**.001	**<**.001	.267	.015	.023	1.000
R34	Adrenaline received^b^	.125	.610	1.000	1.000	1.000	1.000	1.000	1.000	1.000	1.000
R35	AAI prescribed^b^	.011	.286	.512	.401	.729	1.000	1.000	.606	.595	1.000
R36	Both^c^	.345	1.000	1.000	1.000	1.000	1.000	1.000	1.000	1.000	1.000

*Note:* Data are given as no. (%). Statistical significance of differences between patient groups was calculated using Fisher's exact test. Statistically significant *p*-values (*p* < .05) are in bold.

**Abbreviations**: AAI, adrenaline autoinjector; C, column; ColdA, cold-induced anaphylaxis; CWI, complete cold water immersion; N, number of patients; NA, not applicable (ie, no statistics are computed because at least one of the variables is a constant); R, row.

^a^Any cold trigger (i.e., complete cold water immersion, cold ambient air exposure, transition from cold outdoors to warm indoors, localized contact with cold liquids without immersion or ice, and contact with cold surfaces).

^b^Unknown indication(s).

^c^Adrenaline received & AAI prescribed.

